# Heightened Nontesting Risk Mitigation Blood Donor Screening with Retrospective Transcription-Mediated Amplification Testing during a 2023 Autochthonous Florida Malaria Cluster

**DOI:** 10.4269/ajtmh.25-0139

**Published:** 2025-12-09

**Authors:** David J. Sullivan, Marion C. Lanteri, Vanessa Bres, Maesa Hanhan, Marlene Pagan, Alejandro Rey, Korena Thomas, Rita A. Reik

**Affiliations:** ^1^ W. Harry Feinstone Department of Molecular Microbiology and Immunology, Johns Hopkins Bloomberg School of Public Health, Baltimore, Maryland;; ^2^ Creative Testing Solutions, Tempe, Arizona;; ^3^ Department of Laboratory Medicine, University of San Francisco, San Francisco, California;; ^4^ Grifols Diagnostic Solutions Inc., San Diego, California;; ^5^ OneBlood, Inc., St. Petersburg, Florida

## Abstract

Transfusion-transmitted malaria risk in the United States is estimated at less than 1 per 10 million blood donations or about one case every 2 years. Since 2000, the 13 transfusion-transmitted malaria case donations were from former residents of a malaria-endemic area who were mostly outside the deferral windows. Recent autochthonous malaria outbreaks occurred in 2002, 2003, and 2023. From May to early July 2023 in Sarasota County, Florida, symptomatic *Plasmodium vivax* malaria was detected in seven individuals without recent malaria travel history. The local state department of health instituted mosquito control measures and increases in patient malaria syndromic surveillance on May 24th. The local blood center similarly responded to this autochthonous 2023 *P. vivax* outbreak by implementing a nontesting donor risk mitigation strategy of escalating blood donor screening measures and pathogen reduction to minimize the risk of transfusion-transmitted malaria in presymptomatic donors. In the absence of an approved blood donor screening test for malaria at the time, additional nucleic acid testing on 258 donor samples from four Sarasota zip codes and 178 donor samples from eight Miami zip codes collected from July to October was studied retrospectively using a transcription-mediated amplification with a limit of detection ranging from two to seven infected erythrocytes per milliliter. All donor samples were nucleic acid test nonreactive. No local transfusion transmission malaria cases were reported. Both the Florida Department of Health and the blood center took mitigation steps to decrease mosquito and blood donor transmission risks combined with increased surveillance.

## INTRODUCTION

U.S. blood donors represent a healthy population comprising 6.5 million people donating an estimated 11.8 million units of whole blood and red cell products annually.^1^ Presently, the Uniform Donor History Questionnaire and an in-person mini physical are used to screen prospective blood donors.^2^ Additionally, U.S. nonendemic blood donors are deferred within 3 months of travel from malaria-endemic areas. Former residents of malaria-endemic areas and those with recent malaria cases are deferred for 3 years.^3^^,^^4^ Using these measures, there have been only 13 transfusion-transmitted malaria (TTM) cases between 2000 and 2021 (average 0.59/year) or less than 1 in 10 million blood donations in the United States.^5^^,^^6^

Autochthonous malaria outbreaks in the United States are rare. In 2002 in Virginia, two *Plasmodium vivax* cases occurred, and in 2003 in Palm Beach County, Florida, eight *P. vivax* cases occurred.^7^ Twenty years later, seven autochthonous *P. vivax* malaria cases occurred in Sarasota County, Florida.^8^^,^^9^ A single case of *Plasmodium falciparum* was reported in Maryland,^10^ with 2 more *P. vivax* cases reported in Texas^9^ and Arkansas, bringing the total to 10 in 2023 in the United States.^11^ No autochthonous malaria cases were reported in 2024.

**Figure 1.**
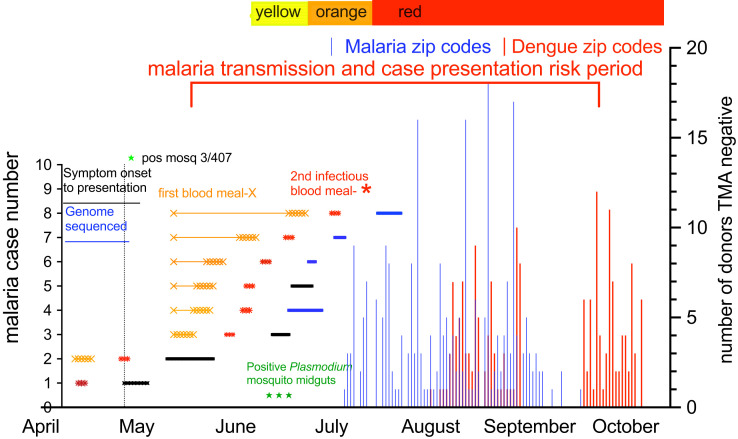
Timeline of *Plasmodium vivax* cases, the range of mosquito feeding for acquisition and transmission, and the risk period for other malaria cases with donor collections and escalation of mitigation strategy. Imported malaria case 1 (April presentation) was infected by a mosquito at least a week before case presentation and was not sequenced. Case 2 (May presentation) had 14 days of previous symptoms, during which this person could have infected other mosquitoes. The mitigation strategy was initiated at Level Yellow at the end of May. The left *y* axis shows the individual malaria cases. The solid black or blue lines represent symptom onset to diagnosis. The blue lines indicate the four cases with identical genome sequencing. The gold X represents the duration range in days of initial mosquito acquisition of *P. vivax* after feeding on case 2 (picking up infectious gametocytes), with subsequent (second) blood feeding transmission of infectious sporozoites a week before clinical case presentation represented by the red asterisk. In the local area, 407 mosquitoes were tested up until August 4, 2025, and 3 mosquitoes collected in early June were positive for *P. vivax* DNA in midguts but negative for infectious sporozoites stages (green stars in early June). The right *y* axis notes the number of retrospective *Plasmodium* test-negative blood donors over 3 months, indicating no measurable contamination of blood supply during the autochthonous outbreak (258 from Sarasota shown as blue vertical lines and 178 from Miami shown as red vertical lines). The time period of maximum malaria blood donor transmission risk was June to August. Monitoring of blood donors went for approximately 10 weeks after the last documented case. Timelines for mosquito feeding and latency are derived from the literature. pos mosq = positive mosquitoes; TMA = transcription-mediated nucleic acid amplification.

In Sarasota, Florida, the malaria cases presented over a 3-month period from April to July 2023, initiating concern for possible TTM risk in presymptomatic blood donors. The details of CDC and Florida Department of Health investigations are briefly summarized here for the context of blood donor supply around possible TTM infection mitigation strategies. The putative index imported malaria *P. vivax* case with symptom onset was initially diagnosed April 20, 2023.^9^ Two weeks later, the first autochthonous *P. vivax* malaria case (homeless status unknown) presented in May with a 14-day symptom history before diagnosis. The local state department of health instituted mosquito control measures and an increase in patient syndromic surveillance for malaria on May 24th. After 2 weeks with no malaria cases, four more autochthonous cases presented in mid-June, with two more autochthonous cases in late June and early July for a 3-month duration 6 weeks after mosquito control measures were started (Figure 1). One of the subsequent six cases was actually detected with syndromic surveillance, with the other five cases reported by providers. The CDC outbreak report noted that three of the *P. vivax* cases were in unhoused people.^9^ To transmit to humans, the mosquito needs to acquire the *P. vivax* infectious gametocytes in a previous blood meal at least 8 days before the transmission bite because of temperature-dependent development processes and relocation from midgut to salivary glands in the mosquito.^12^ The prepatent time (time to detectable parasitemia by microscopy from infected mosquito bite) is approximately 12–14 days in *P. vivax* case presentations.^13^^–^^16^ *Plasmodium vivax* gametocytes take just 2 days to develop, they last in blood circulation for another 3 days, and they even appear within a week after mosquito inoculation, which includes the liver stage.^17^ The time from an infectious bite transmitting to mosquito stages to detectable parasitemia can be 3–4 weeks, which is important for strategies to contain transmission and protect the blood supply. Timelines for mosquito feeding and latency indicated in Figure 1 are proposed from the literature. These were not established by data in this study. In a genome sequence analysis previously published by the University of South Florida working with the Florida Department of Health, malaria cases 4, 6, 7, and 8 who presented as the last four cases were identical or clonal.^18^ The initial *P. vivax* imported case and case 2 did not have evaluable DNA material. The CDC investigated 30 additional people with syndromes suggestive of malaria, but all were diagnosis test negative with the BinaxNOW^TM^ Malaria (Abbott, Scarborough, Maine) point-of-care rapid diagnostic tests, which have a sensitivity of 10,000–100,000 per 1 mL of blood.^9^ In the local area, 407 mosquitoes were tested until August 4, 2023. Three mosquitoes collected in early June were positive for *P. vivax* DNA in midguts, meaning that they had fed on persons with blood-stage *P. vivax*, but all were negative for infected salivary gland sporozoites stages representing the development to an infectious mosquito to humans.^9^

Separate from the mosquito control interventions and increased vigilance for febrile patient presentations, here we describe the operational strategies implemented by a large regional blood center (BC) serving Florida and surrounding states to mitigate asymptomatic donor transfusion-transmission risk from *Plasmodium* species (malaria). These strategies are applicable to other mosquito-borne pathogens, including dengue virus, chikungunya virus, Zika virus, and yellow fever virus. Given the absence (at the time in 2023) of licensed donor screening tests, the BC developed a multitiered, risk-based donor screening and product management protocol to protect the safety of the blood supply. In addition, 489 blood donor samples were retained in the summer of 2023 (305 from malaria-impacted zip codes and 184 from dengue-impacted zip codes), with 436 tested with retrospective *Plasmodium* nucleic acid testing aimed at identifying possible subclinical infections at the time of donation.

## MATERIALS AND METHODS

### Blood donor pathogen mitigation strategy.

After notification by the Florida Department of Health of autochthonous malaria confined to a specific location in May, the BC implemented its Vector-Borne Pathogen Mitigation Strategy (VPMS), which consists of heightened donor education and screening measures, product embargoes, and donor call backs along with pathogen reduction of eligible products (INTERCEPT^TM^ for Platelets and Plasma, Cerus Corporation, Concord, CA) for blood donors in the affected area. The BC escalated the VPMS to Level Yellow on May 27, 2023 for 3 weeks and Level Orange on June 20, 2023 for 2 weeks followed by Red Level on July 5, 2023 for 14 weeks. The VPMS has been successfully used by the BC since 2013 during outbreaks of dengue, chikungunya, and Zika, with additional malaria modifications.

The mosquito-borne pathogen risk mitigation strategy includes classification of geographic regions by case burden and implementation of corresponding donor and product management procedures. Counties are designated as follows. An affected region has one or more confirmed locally acquired cases of a mosquito-borne pathogen. An adjacent region is a county bordering an affected region with no known locally acquired cases. Each affected region is assigned a response level (Green, Yellow, Orange, or Red) based on disease activity and case load (Table 1). Donor interventions are scaled accordingly.

**Table 1:** Florida blood center Vector-Borne Pathogen Mitigation Strategy including malaria

Region Status	Add Educational Materials	Add UDHQ Questions	Tag Units for PRT	7-Day Product Quarantine Release If No PDI Received	14-Day Product Quarantine with Mandatory Donor Call Back Required for Release
Adjacent region*	Yes	NA	NA	NA	NA
Yellow: Travel associated and >1 autochthonous case in a county in a rolling 30-day (virus) or 70-day (malaria) period	Yes	Yes	NA	NA	NA
Orange: 2–5 autochthonous cases in a zip code in a rolling 30-day (virus) or 70-day (malaria) period	Yes	Yes	Yes	Yes	NA
Red: >6 autochthonous cases in a zip code in a rolling 30-day (virus) or 70-day (malaria) period	Yes	Yes	Yes	NA; released at day 14	Yes

NA = not applicable; PDI = postdonation information; PRT = pathogen reduction technology; UDHQ = Uniform Donor History Questionnaire.

* Adjacent region has no autochthonous cases.

When Level Yellow is declared in an affected region, donors are provided a standardized educational guide customized for the vector and pathogen of interest. Collection staff members initiate targeted mosquito-borne pathogen questioning (Table 2). Adjacent regions implement the standardized educational guide. At Level Orange, daily enhanced biovigilance through Enterprise Business Intelligence reports identifies at-risk donations. Donors are asked the additional mosquito-borne pathogen questions at collection. Educational information is provided to all donors in the affected zones. Platelets-pheresis products collected in affected zones are flagged for pathogen reduction. Blood products that cannot be pathogen reduced and are collected in affected zones are quarantined for 7 days, and donor contact (e-mail, voicemail, or live conversation) is initiated on day 1 or 2 after collection. Those products are released after donor initial contact has been made and no postdonation symptoms are reported by the donor within 7 days of donation. Blood products from donors to whom no initial contact could be made (no voicemail or e-mail available) were discarded. If postdonation symptoms are reported, the donor is deferred, and collected units are discarded.

**Table 2:** Questionnaire for mosquito-borne pathogen at collection

Questionnaire
Educational information
**Thank you for your donation today.** We want you to know information that is important for patients who may receive your blood and that may also be important to your own health. The Department of Health has reported locally acquired cases of mosquito-borne diseases such as dengue fever, chikungunya, zika fever, yellow fever and malaria. These diseases are transmitted to humans by infected mosquitoes and cause mild to severe flu-like illness that on occasion can be serious enough to result in death. Although you reported feeling completely well and healthy at the time of your donation, you may still have been infected with one of these mosquito-borne pathogens and not feel anything. In this situation, you may develop symptoms in the coming weeks. PLEASE notify us as soon as possible at 1.888.###.####) if in the next 4 weeks you experience. Intermittent attacks of shivering and chills, followed by a high fever, then sweating and a return to normal temperature or 2 or more of the following symptoms: Fever ≥100°F, Muscle and/or joint aches or weakness or fatigue, Nausea and vomiting, Rapid heart rate and rapid breathing, Headache, Eye pain, A rash, Bleeding or easy bruising. **By notifying us, you may prevent the blood that you donated today from being used and possibly infecting a patient receiving a transfusion.**
Additional donor collection screening questions
In the past 4 weeks, have you experienced: Intermittent attacks of shivering and chills, followed by a high fever, then sweating and a return to normal temperature or 2 or more of the following symptoms: Fever ≥100°F, Muscle and/or joint aches or weakness or fatigue, Nausea and vomiting, Rapid heart rate and rapid breathing, Headache, Eye pain, A rash, Bleeding or easy bruising.
In the past **4 weeks**, has someone in your household or workplace had Dengue Fever, Chikungunya Fever, Zika Fever or Malaria?

At Level Red, all prior Level Orange actions continue with initial donor contact on day 7 or 8. Products are quarantined for 14 days rather than 7 days. Donor contact follow-up uses Red Scripts for communication: “If you continue to feel healthy and well, PLEASE call back to confirm, so we may use your donation.” Donations lacking donor callback on or after day 7 or containing high-risk exposure are discarded unless pathogen reduced. Cessation of questioning and educational guide distribution is only after the expiration of the monitoring window and in the absence of new cases. The monitoring period for malaria was arbitrarily and conservatively set at 10 weeks from the last confirmed case or infected mosquito detection, which can be difficult in real time to validate. Once a region enters Level Yellow, reversion to Level Green is not permitted before November 1, regardless of the monitoring outcome.

### Retrospective testing of collected samples.

The donor study protocol was approved by the Institutional Review Board Advarra (protocol no. Pro00079961). Donors were initially screened and accepted using the nontesting VPMS (Table 1). The 436 tested donors were from a pool of 489 eligible donors, of whom 440 consented for future infectious diseases testing and 436 were tested for malaria (258 donated from July 5 to September 17 in four Sarasota zip codes and 178 donated from August 1 to October 6 during and after a dengue outbreak from the nonmalaria Miami area) (Table 3). Whole-blood samples from 436 blood donors were collected, and individual whole-blood lysates were manually prepared by mixing 0.9 mL of fresh whole blood with 2.7 mL of Parasite Transport Media, a proprietary lysis and RNA-stabilizing solution (Grifols Diagnostic Solutions Inc., San Diego, CA) in an individual donor lysate (IDL) tube. Individual donor lysates were then frozen at ≤−20°C until testing was performed on 0.5 mL IDL from the initial 0.9 mL blood input. Samples were retrospectively tested on the investigational Procleix *Plasmodium* Assay (Grifols Diagnostic Solutions Inc.), a qualitative in vitro transcription-mediated nucleic acid amplification test for 18S ribosomal RNA from five *Plasmodium* species (*P. falciparum*, *Plasmodium ovale*, *P. vivax*, *Plasmodium malariae*, and *Plasmodium knowlesi*). This assay is not yet available for blood donor screening in the United States, but it is available in countries/regions recognizing the Conformité Européenne mark. The assay is conducted on a fully automated Procleix Panther System (Grifols Diagnostic Solutions Inc.). Specimens with an analyte signal/cutoff ≥1.00 are considered reactive.^19^ The assay does not provide species-level determination, only genus-level *Plasmodium*.

**Table 3:** *Plasmodium* testing donor numbers and percentages

Total Risk	Donors	*Plasmodium* Tested	Percentage of Donors Tested	Total Population	Donor Percentage of Total Population	Tested Percentage Population	Number of Zip Codes
Total malaria risk	305	258	85	82,854	0.37	0.31	4
Total dengue risk	184	178	97	282,529	0.07	0.06	8
Total	489	436	89	365,383	0.13	0.12	12

## RESULTS

### Mitigation strategy.

The first locally acquired cases of *P. vivax* malaria since 2003 were identified in Sarasota and Manatee Counties. In response, OneBlood, Inc. (St. Petersburg, FL) escalated to VPMS Level Yellow on May 27, 2023 for 3 weeks and Level Orange on June 20, 2023 for 2 weeks followed by Level Red on July 5, 2023 for 14 weeks (Figure 1; Table 1), which included donor collection questioning (Table 2), product quarantine until postdonation contact after 14 days, and follow-up protocols. Less than 3% of collected products were discarded under Level Orange. In the Level Red period, approximately 60% of products were discarded. No malaria-related transfusion transmitted infections were reported.

Throughout these outbreaks, OneBlood’s tiered response system was activated per predefined disease burden and geographic risk. Across all events, donor notification, pathogen reduction technology (PRT) for platelets, and follow-up compliance were >95% within 7 days of donation for Level Orange passive callback for positive symptoms. Product discards at Level Orange were minimized through early quarantine release after negative postdonation information processes. The more active mandatory donor callback, where the donor must return information to the BC, reflects a drop in release and increase in discards for Level Red through October. Blood center interventions were associated with no TTM, although a causal effectiveness cannot be inferred from the small sample size without randomization.

### Donor malaria testing.

The 258 malaria Sarasota area donors from a pool of 305 donors had blood collected from July 5 to September 17, with the last 4 collected after September 5 or 2 months after initiation of collection (Table 3). The 178 dengue area collections more than 200 miles away in Miami, Florida from a pool of 284 donors were from August 1 to October 6, with a 6-week overlap from August 1 to September 17 with malaria collections. The malaria area collections were from four zip codes with a total population of approximately 83,000 people, and the dengue collection was from eight Miami area zip codes representing about 365,000 in population. During the donor collection period, 85% of the donors from malaria risk areas were tested, representing only 0.3% of the total population. The “negative” malaria control collections from the dengue outbreak represented 97% of the donors during those time periods but only represented 0.1% of the total population. They were eligible donors as well as asymptomatic (i.e., not febrile) and passed heightened blood donor screening per the BC VPMS, and their eligible products were pathogen reduced as indicated and released into the blood supply. These samples were tested retrospectively with the Procleix *Plasmodium* Assay for the detection of panspecies *Plasmodium* 18s rRNA present in thousands of copies per cell. Of 436 samples, 5 samples had an initial invalid result, of which 4 samples were valid upon retest and 1 sample was invalid from the dengue group. The overall final validity was 99.8% (*n* = 435/436). All 435 samples with a valid result collected in the local area from July 2023 to October 2023 were nonreactive for *Plasmodium* (Figure 1).

## DISCUSSION

This study highlights the strategy of a structured, scalable risk mitigation framework for protecting the blood supply from mosquito-borne pathogens in a subtropical U.S. region prone to arboviral and parasitic disease transmission. By aligning interventions with real-time public health data and implementing escalation protocols based on local case counts and geographic proximity, the BC was able to ensure transfusion safety while minimizing unnecessary donor deferral or product loss. The BC measures designed to reduce transfusion-transmitted infection are separate from vector control measures and increased patient surveillance to stop an ongoing infectious diseases outbreak.

The adaptive BC response model—reviewed by regulatory bodies and industry stakeholders—showcases a practical and reproducible method for regional BCs facing vector-borne threats. Notably, the tiered system enabled nuanced, zip code-level interventions with minimal service disruption, preserving platelet availability through PRT and reducing discard rates with efficient donor follow-up mechanisms. Limitations include reliance on public health case reporting accuracy and timeliness as well as potential underdetection of asymptomatic infected donors. Another limitation was donor active engagement to BC communications for Level Red, during which more loss of product was noted because of donor failure to return communication to BC. However, despite no transfusion-transmitted cases reported, the effectiveness of the strategy was not able to be measured in this noncontrolled real-world analysis.

Blood donor serologic testing has been advocated for infectious disease screening for respiratory pathogens and vector-borne diseases, like Chagas, whereas nucleic acid testing is approved for HIV, hepatitis B virus, hepatitis C virus, Babesia, West Nile Virus, and Zika.^20^ The risk of TTM is lower than 1 in 1 million. Although this study still does not address sensitivity, specificity, or predictive values of *Plasmodium* NAT detection, this study involved some of the largest numbers of donor samples tested in the setting of autochthonous *P. vivax* outbreaks in the United States.

In reviewing the tempo and location of the autochthonous cases observed in the 2023 Florida malaria cluster, we hypothesize that the second autochthonous case infected subsequent mosquitoes and initiated the genetically identical infections in cases 4, 6, 7, and 8 occurring in people up to the first week of July. If the last case was similarly infectious as the second through eighth cases, the risk period for malaria in the blood supply could be predicted to primarily extend into September. Evidence to support the genetic relatedness of cases 2–8 is from the 2002 autochthonous *P. vivax* cases, which were genotypically identical, indicating a common source.^21^ In the 2003 Palm Beach, Florida outbreak, all seven *P. vivax* cases were genetically identical by multilocus genotyping of microsatellites.^22^ Although there is not direct laboratory evidence that the other three cases were identical to the other four in 2023 in Sarasota, Florida, given the context of the 2002 and 2003 outbreaks, the probability is greater for the genetic relatedness of the seven cases in 2023 in relationship to the probability of a separate introduction from another source occurring twice in the same location in an event happening about every 10 years among 300 million people in the malaria hypoendemic United States. The relevance to donors being infected but asymptomatic with *P. vivax* malaria is the now real possibility of a blood donor becoming symptomatic after donation. The BC did not screen every donor, which was not feasible; a priori, there was no knowledge of how long the ongoing outbreak would last. In hindsight, the overlapping risk in time from the last case presentation in July to collection is only a few weeks, but this was not known at the initial setup of collections.

The Florida Department of Health began enhanced surveillance for malaria-infected mosquitoes and enhanced mosquito control measures on May 24, including aerial and ground spraying for adult mosquitos and a variety of larvicide applications, including to large wetland areas. These measures most likely had the greatest impact on stopping the outbreak.

Separate from controlling the autochthonous outbreak, there existed new increased risks to the blood supply from preclinical healthy donors having malaria symptoms a few days after donation. The BC also instituted enhanced surveillance and mitigation control measures to ensure prevention of TTM. If a mosquito bit an infectious host, there is a 1- to 2-week lag before a transmitting bite to a new host followed by a 4-week time period to case presentation (90% of travelers have primary malaria case presentation within 4 weeks) or about 6 weeks. The 10-week window arbitrarily and conservatively adds another 4 weeks.

A total of 18 autochthonous malaria cases (17 owing to *P. vivax* and 1 owing to *P. falciparum*) have been recorded in the United States since 2003.^6^^,^^23^ During the past 20 years, there have been only 13 cases of TTM: 9 *P. falciparum* cases, 2 *P. malariae* cases, and 2 *P. ovale* cases, with no *P. vivax* cases.^24^^,^^25^ Additionally, all traced donors were former residents of malaria-endemic Africa.^4^ None were U.S. citizens traveling back from malaria-endemic areas. This contrasts with the 93 TTM cases from 1963 to 1999: 33 *P. falciparum* cases, 25 *P. vivax* cases, 25 *P. malariae* cases, and 5 *P. ovale* cases.^26^ The country of origin was determined in 64 of 93 implicated donors, of which 38 of 64 (59%) were foreign born and 26 were born in the United States. In this group, the longest time from travel to a malaria region and donation of malaria-infected blood was 44 years in the case of *P. malariae*, 5 years in the case of *P. falciparum*, 7 years in the case of *P. ovale*, and 2.5 years in a case of *P. vivax*, with most outside of the deferral interval of 3 years.^26^ In that last century time period, only 3 donors were U.S. citizens returning from recent travel (23 other United States-born donors were prolonged residents in malaria-endemic countries), and subsequently, they became infected blood donors. From 1963 to 2000, recent travelers were deferred for 6 months. However, those who took malaria chemoprophylaxis were deferred for 1–3 years depending on changing guidelines, and military residents were deferred for 3 years. The definition of a malaria residence versus longer travel stay was not clearly defined during this time period. Ten of the 93 TTM recipients died of severe malaria.^26^

Preparedness for emerging infectious disease outbreaks is one of the strategic initiatives undertaken by the BC to maintain the safety and availability of the blood supply during an autochthonous vector-borne agent outbreak. Indeed, the BC is mostly collecting blood in Florida, a U.S. state known to be impacted by local outbreaks of mosquito-borne agents. Past Florida experience includes responses to outbreaks of dengue, chikungunya, and Zika viruses as well as malaria. Close collaboration with the local health department and CDC has allowed for appropriate communication for optimum reactivity to local emerging threats. As vector-borne outbreaks are unpredictable, the BC developed the VPMS, which was used successfully several times over the past 12 years, including deployment during the 2023 malaria cluster. Although the VPMS has proven to be effective to date, with no cases of transfusion-transmission infections associated with its use during outbreaks in Florida, concerns remain regarding subclinical blood donor infections. Similarly, routine donor follow-up after donation, such as reporting medical symptoms to blood bank staff, only partially addresses the risk associated with donations collected during the presymptomatic phase of infection because of variability in symptom development times.

Another mitigation strategy used by the BC during clusters and outbreaks is the use of an approved amotosalen and ultraviolet light pathogen reduction (PR) system for the inactivation of infectious agents in platelets and plasma.^27^ Such a system has been in use at the BC since 2016. A different nonapproved PR with amustaline and glutathione that is still in development adequately inactivates erythrocytic *Plasmodium* among other species.^28^ Because of antibodies to acridine (a by-product of amustaline) found in transfused patients, this technology is not approved in the United States.^29^

This analysis involved an outbreak where at least three of the individuals experienced underhousing/homelessness; this might lead to different risks for vector-borne infection transmission from those in adequate housing, which may be more or less representative of the blood donor population. During a malaria outbreak, housing risk for vector-borne disease might be incorporated into immediate blood donor screening policies.

The Food and Drug Administration (FDA) issued draft guidance to reduce the risk of TTM in January 2025 after the FDA approval of the Cobas malaria test (Roche, Branchburg, NJ) in 2024.^30^ The selective testing strategy considers testing each donation from those donors with previous malaria, one-time testing of donors with prior residence in malaria-endemic regions and donors who have traveled to malaria-endemic regions in past 12 months, and testing all donations collected in regions with autochthonous malaria outbreaks.^30^ Although the availability of testing may seem to make testing a practical approach, there are limiting factors to consider. Testing increases the cost of the blood product, and this cost is passed on to the hospitals, thus increasing health care costs. In instances where outbreaks are small, focal, and time limited (as in this case), the complexity and cost of bringing up testing in the impacted areas have to be considered. By the time that the test can be brought up in the blood computer system, the outbreak may be over. Another consideration is that the test manufacturers may not be able to guarantee that tests will be available on demand for unexpected small outbreaks as they have constraints around the production of small volumes of tests for random events. Testing all donors throughout the year is the model that test manufacturers are able to best accommodate. With small clusters occurring only every 20 years, large-scale, ongoing testing of donors for malaria in focal regions of the United States appears to not be cost effective.

## CONCLUSION

With the implementation of the VPMS and the containment efforts of the Florida Department of Health, the blood supply was not significantly impacted by the autochthonous outbreak as demonstrated by no TTM. From a BC perspective, the highly sensitive Procleix *Plasmodium* Assay offers the opportunity to enhance blood safety during autochthonous malaria outbreaks. The blood donor population testing for infectious diseases represents a small fraction of the total population and is unable to measure total population malaria risk or contribute to infection outbreak control. Even though this study tested a few hundred donors, the blood donor testing extent, duration, and specific zip codes within a region with autochthonous malaria all remain as unanswered questions in the context of infrequent malaria outbreaks, which may include areas like Maryland and Arkansas with more limited BC mitigation strategies available.
